# Early hematological parameters as predictors for outcomes in children with dengue in northern India: A retrospective analysis

**DOI:** 10.1590/0037-8682-0519-2020

**Published:** 2021-01-29

**Authors:** Sumi Nandwani, Bhanu Kiran Bhakhri, Nupur Singh, Ruchi Rai, Dharmender Kumar Singh

**Affiliations:** 1 Superspecialty Pediatric Hospital & Postgraduate Teaching Institute, Department of Microbiology, Noida, Uttar Pradesh, India.; 2 Superspecialty Pediatric Hospital & Postgraduate Teaching Institute, Department of Pediatrics, Noida, Uttar Pradesh, India.; 3 Superspecialty Pediatric Hospital & Postgraduate Teaching Institute, Department of Neonatology, Noida, Uttar Pradesh, India.

**Keywords:** Hemorrhagic fever, Leucocyte count, Mean platelet volume, Platelet count, Mortality predictors

## Abstract

**INTRODUCTION:**

Dengue presents with a variable clinical course, ranging from mild illness to potentially fatal hemorrhage and shock. We aimed to evaluate the capabilities of various hematological parameters observed early in the course of illness for predicting the clinical outcomes of illness.

**METHODS:**

We retrospectively analyzed the records of children admitted in the pediatric inpatient services of the institute with dengue between 2017 and 2019. We determined the relationships between the hematological parameters observed during the first evaluation and the various clinical outcomes.

**RESULTS:**

We evaluated data from 613 patients (age range, 26 days to 17 years). Of these, 29.85% exhibited fever with warning signs, and 8.97% had severe dengue. Lower values of hemoglobin, platelet count, mean corpuscular volume, mean corpuscular hemoglobin concentration, and mean platelet volume, and higher values of total leukocyte count (TLC), hematocrit, and red cell distribution width variably correlated with numerous clinical outcomes-duration of hospital stay, development of complications, requirement of blood component transfusion, inotropic support, and mortality. Among the parameters, TLC ≥20,000/mL and initial platelet count ≤20,000/mL significantly associated with mortality, with odds ratios (95% confidence interval) of 11.81 (4.21-33.80) and 5.53 (1.90-16.09), respectively.

**CONCLUSIONS:**

Hematological parameters observed early during dengue infection may predict its clinical outcomes in infected children. Initial high TLC and low platelet count are potential predictors of fatal outcomes in the course of disease.

## INTRODUCTION

For the last three decades, dengue has continued to pose a major public health problem worldwide. Globally, there are approximately 100 million infections reported each year with up to 2% resulting in a fatal outcome^1^. These outbreaks persistently challenge regional health systems and the economy, particularly in developing countries where they are more prevalent. The economic impact of dengue in India in 2006 was estimated to be USD 27 million^2^.

Dengue targets people from a wide range of sociodemographic characteristics and contributes to considerable morbidity and mortality in the pediatric population^3^. It presents with a spectrum of clinical courses among children, ranging from minor insignificant illness to potentially fatal hemorrhage and shock^3^. Globally, researchers are striving to unearth the determinants of its anticipated clinical course to utilize the available resources with maximum cost effectivity. The proposed determinants range from certain clinical features^4^ along with common laboratory parameters^5^ to specific biomarkers and gene expression^6^ and have variable clinical applicability. Among the hematological parameters, hemoconcentration and thrombocytopenia markers are the most widely studied and utilized for clinical decisions^7^. 

As hematological derangements are among the most frequently observed manifestations in severe dengue infection, we theorized that detailed exploration of hematological parameters, including red cell indices and platelet size, observed early in the course of illness might predict various clinical outcomes. Being commonly available and routinely performed as part of investigations, these predictive factors could assist clinicians in resource-limited settings to identify children with anticipated severe illness. 

## METHODS

This study was conducted at a tertiary care pediatric teaching hospital in northern India. We retrospectively analyzed the records of children with laboratory-confirmed (NS1 antigen or anti-dengue IgM antibody) dengue infection, admitted between 2017 and 2019. The study protocol was approved by the institutional ethics committee (No 2019-01-IM-01). We recorded various parameters-(1) demographic: age, sex; (2) clinical: weight for age, presenting symptoms, signs observed during first examination, duration of illness at first blood sampling, and duration of illness at admission; and (3) hematological parameters observed in the first blood sample: hemoglobin level (Hb), total leucocyte count (TLC), hematocrit (Hct), platelet count, mean corpuscular volume (MCV), mean corpuscular hemoglobin concentration (MCHC), red cell distribution width (RDW-CV), and mean platelet volume (MPV). The minimum platelet count during the illness was also documented. The weight for age was recorded as ‘z’ scores according to the Indian Academy of Pediatrics anthropometric data. For the hematological parameters, we used reports generated by the 5-part automated analyzer, CELL-DYN Ruby System (Abbott Core Laboratory, Chicago, IL, USA). We extracted various clinical outcomes, including duration of hospital stay, requirement of packed red blood cell (PRBC) or platelet transfusions, need for inotropic support, occurrence of complications, and mortality, from patient records. 

The observations were entered into Microsoft Excel (Microsoft, Redmond, WA, USA), and statistical analysis was performed using the SPSS Statistics software (IBM, Armonk, NY, USA). For descriptive statistics, continuous measures were presented as means and standard deviations (SD), while categorical variables were presented as absolute and relative frequencies. Parameters were compared using ‘z’ statistics. Correlations among various parameters were explored using Pearson’s coefficient of correlation. Multivariate analysis was performed for various hematological parameters to calculate odd ratios (with 95% confidence intervals (CI)) for risk of various dichotomous clinical outcomes. The distribution of relevant parameters was graphically depicted using boxplots. For parameters significantly associated with mortality, logistic regression and receiver operating characteristic (ROC) curve analyses were performed to identify their cut-off levels for predicting mortality. Taking reference from the observations of Malhi et al.^8^ and considering 17,000/mL as the mean difference of platelet count among uncomplicated and complicated dengue cases, a sample size of 448 was required for the study with 80% power**.**

## RESULTS

A total of 1195 samples were reported to be positive for dengue (NS1 antigen or anti-dengue IgM antibody) during the 3-year study period. Details from 613 admitted patients were available for analysis following retrieval from the record repository. Table 1 presents their baseline characteristics. The age of patients ranged from 26 days to 17 years. The weight for age of the group was below the national average. The majority of patients presented with fever, vomiting, and abdominal pain. Overall, 38% of the cases were positive for both NS1 antigen and anti-dengue IgM antibody; however, the remaining cases were positive for only one of the two. Twenty patients were diagnosed with malaria coinfection. Based on the presentation and clinical course, 183 (29.85%) patients exhibited dengue fever with warning signs, and 55 (8.97%) had severe dengue. 

**TABLE 1:** Baseline characteristics of patients infected with dengue (n = 613).

Characteristic	Mean (SD)	Frequency	
		n	%
Male sex		374	61
Symptoms/signs at presentation			
Fever		608	99.2
Vomiting		313	51.1
Pain abdomen		253	41.3
Bleeding from any site		163	26.6
Jaundice		13	2.1
Palpable liver or spleen		243	39.6
Positive NS1 antigen		370	60.3
Positive anti-dengue IgM antibody		476	77.7
Coexisting malaria (smear or antigen positivity)		20	3.26
Age (years)	7.14 (4.26)		
Weight for age (z score)	−1.17 (5.54)		
Duration of symptoms at first hematological evaluation (days)	4.24 (1.43)		
Duration of symptom at admission (days)	4.85 (1.71)		
Hemoglobin level (g/dL)	11.53 (2.17)		
Total leucocyte count (per mL)	7470 (6054.17)		
Platelet count at presentation (100,000 per mL)	0.95 (0.85)		
Minimum platelet count during illness (100,000 per mL)	0.72 (0.34)		
Mean corpuscular volume (fL)	77.02 (9.42)		
Mean corpuscular hemoglobin concentration (g/dL)	31.93 (2.39)		
Mean platelet volume (fL)	11.17 (2.31)		
Red cell distribution width (%)	14.23 (1.44)		
Hematocrit (%)	39.01 (7.06)		

At the initial evaluation, 28 (4.56%) patients had Hb level ≤8 g/dL. The TLC was ≥10,000/mL and ≥20,000/mL in 122 (19.90%) and 25 (4.07%) patients, respectively. Most of the patients had leucopenia. The initial platelet count was ≤20,000/mL among 37 (6.03%) patients, while only 3 (0.5%) children had platelet count ≤10,000/mL. During the course of illness, 97 (15.82%) children had a minimum platelet count falling below 20,000/mL. In the first sample, 120 (19.57%) children showed hemoconcentration with Hct ≥45% and 116 (18.9%) had MPV ≤9 fL. Overall, the group showed low MCV and MCHC and high RDW-CV. Table 2 summarizes the clinical outcomes among the cases. Complications in the form of azotemia and effusions in serous cavities were each observed in approximately 3% of patients. 

**TABLE 2:** Clinical outcomes of patients with dengue (n = 613).

Clinical outcomes	n	%
Requirement of platelet transfusion	170	27.73
≤2 units	36	5.87
3-5 units	101	16.47
≥6 units	33	5.38
Requirement of packed red blood cell transfusion	30	4.89
Requirement of inotropic support	28	4.7
Complications		
Azotemia	18	2.93
Peritoneal bleeding	1	0.16
Pleural effusion +/− ascites	17	2.77
Duration of hospital stay in days, mean (SD)	5.53 (1.57)	
Mortality	21	3.42

The relationship between sex and mortality was not significant. We observed a statistically significant correlation between occurrences of any type of complication and sex. There was no significant difference in mean weight for age among the two sexes. It was not associated with any other clinical outcome. 

**FIGURE 1:**
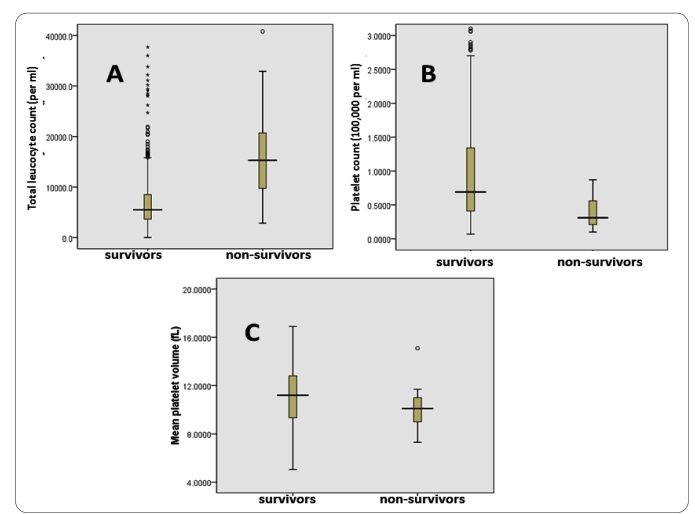
Distribution of parameters significantly related with mortality (**A:** total leucocyte count, **B:** initial platelet count, **C:** mean platelet volume) among survivors and non-survivors.

Bivariate correlation analyses revealed no significant correlation between age or weight for age and any of the clinical outcomes. History of bleeding from any site and presence of jaundice on examination significantly correlated with duration of hospital stay, requirement of platelet transfusions, need for inotropic support, and mortality. The presence of jaundice also significantly correlated with the requirement of PRBC transfusion during the clinical course. Palpable liver or spleen significantly correlated with the development of complications in children. Among the hematological parameters, the requirement of PRBC transfusion correlated with all studied parameters except minimum platelet count and MCV. While initial platelet count showed a significant inverse correlation with most outcomes, the minimum platelet count during hospital stay failed to associate with any outcome. Higher TLC significantly correlated with unfavorable results for all clinical outcomes. The MPV showed a significant inverse relationship with all outcomes except the duration of hospital stay. Both mortality and requirement of inotropic support significantly correlated with TLC, initial platelet count, and MPV. Among the survivors and non-survivors, the (mean; SD) TLC (per mL, 7,077; 5,324 vs. 18,320; 12,272), initial platelet count (100,000 per mL, 0.97; 0.85 vs. 0.38; 0.21), and MPV (fL, 11.21; 2.32 vs. 10.12; 1.69) were significantly different (p<0.05), and the distribution is presented in Figure 1. 

**FIGURE 2:**
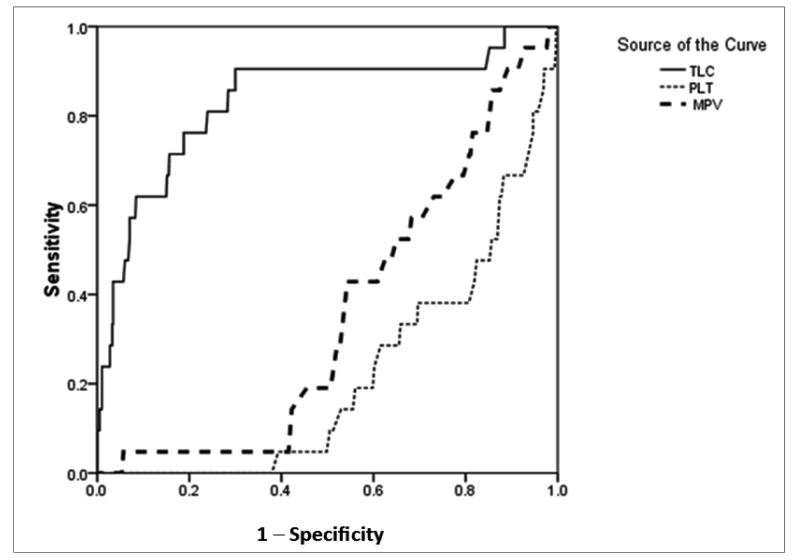
ROC curve analysis for the prediction of mortality using the area under the curve (AUC) for total leucocyte count (TLC), initial platelet count (PLT), and mean platelet volume (MPV).

Table 3 displays the results of multivariate analysis estimating the odds ratios (with 95% CI) of various hematological parameters for the risk of various dichotomous clinical outcomes. For mortality as an outcome, the odds ratios (95% CI) for TLC ≥20,000/mL, initial platelet count ≤20,000/mL, and MPV ≤9 fL were 11.81 (4.21-33.80), 5.53 (1.90- 16.09), and 1.71 (0.65-4.50), respectively. Hence, the association between MPV and mortality could not be established. For the prediction of mortality using ROC curve analysis (Figure 2), the area under the curve (AUC) for TLC, initial platelet count, and MPV were 0.83 (p<0.01), 0.22 (p<0.01), and 0.36 (p<0.05), respectively. From the ROC curve, the initial TLC >7,500/mL provided a cut-off level for predicting mortality among children with dengue infection with 90% sensitivity and 70% specificity. From the respective ROC curves, the initial platelet count ≤15,000/mL and MPV <8 fL provided sensitive cut-off values for mortality, albeit with very poor specificity. Multivariate logistic regression revealed the effect of all hematological parameters on the risk of death from dengue infection. The regression model was statistically significant (χ^2^ −49.62, p<0.05), explained 30% (Nagelkerke R^2^) variance in the risk of mortality, and correctly classified 96% of the cases. Higher TLC along with lower platelet count, Hb level, and Hct level were associated with higher likelihood of mortality.

**TABLE 3:** Multivariate analysis showing the risk of dichotomous clinical outcomes with various hematological parameters.

	Development pleural effusion +/− ascites	Duration of hospital stay ≥8 days	Requirement of platelet transfusion	Requirement of red blood cell transfusion	Requirement of inotropic support	Mortality
Hemoglobin ≤8 g/dL	**7.49**	**6.65**	1.79	**58.08**	2.74	0.96
	**(2.26-24.76)**	**(2.71-16.29)**	(0.81-3.94)	**(22.84-147.65)**	(0.77-9.71)	(0.95-1.00)
Total leucocyte count ≥20,000/mL	1.46	**3.62**	2.06	**7.25**	**7.94**	**11.81**
	(0.18-11.43)	**(1.29-10.20)**	(0.91-4.62)	**(2.66-19.80)**	**(2.88-21.83)**	**(4.21-33.80)**
Initial platelet count ≤20,000/mL	**5.30**	2.29	**53.50**	**5.68**	**7.77**	**5.53**
	**(1.63-17.17)**	(0.84-6.23)	**(12.68-225.60)**	**(2.25-14.31)**	**(3.15-19.18)**	**(1.90-16.09)**
Minimum platelet count ≤20,000/mL	**3.94**	**4.17**	**55.29**	**3.91**	**8.32**	7.98
	**(1.46-10.62)**	**(2.15-8.07)**	**(25.82-118.39)**	**(1.81-8.42)**	**(3.79-18.28)**	(0.96-19.54)
Mean corpuscular volume ≤70 fL	**4.84**	1.90	1.37	**5.18**	**2.36**	1.65
	**(1.82-12.82)**	(0.96-3.78)	(0.89-2.10)	**(2.45-10.95)**	**(1.06-5.25)**	(0.62-4.35)
Mean corpuscular hemoglobin concentration ≤30 g/dL	**0.03**	**2.59**	1.35	**7.10**	1.83	1.26
	**(1.09-8.40)**	**(1.29-5.19)**	(0.85-2.15)	**(3.33-15.12)**	(0.76-4.44)	(0.42-3.84)
Red cell distribution width ≥16%	2.29	**2.44**	1.28	**3.39**	1.21	1.21
	(0.72-7.23)	**(1.15-5.21)**	(0.76-2.16)	**(1.48-7.72)**	(0.41-3.60)	(0.35-4.22)
Hematocrit ≥45%	1.73	1.29	**2.32**	**0.13**	1.68	0.96
	(0.60-5.00)	(0.62-2.71)	**(1.52-3.52)**	**(0.02-0.99)**	(0.72-3.90)	(0.32-2.90)
Mean platelet volume ≤9 fL	**11.18**	**2.50**	**1.85**	2.21	**2.91**	1.71
	**(3.85-32.43)**	**(1.28-4.87)**	**(1.21-2.83)**	(1.00-4.87)	**(1.32-6.40)**	(0.65-4.50)

All values are presented as odds ratio (95% confidence interval). Significant figures are marked as bold.

## DISCUSSION

The retrospective analysis of 613 patients admitted with dengue infection showed variable derangements in clinical and laboratory parameters. The female sex experienced a significantly higher number of complications. History of bleeding from any site and presence of jaundice or palpable liver or spleen was associated with various poor outcomes. The lower values of Hb, platelet count, MCV, MCHC, and MPV and higher values of TLC, Hct, and RDW-CV correlated with various clinical outcomes, such as duration of hospital stay, development of complications, requirement of blood component transfusion, inotropic support, and mortality. Among the observed hematological parameters, higher TLC at presentation emerged as the most important predictor for mortality. 

As our institute caters to children from a wide spectrum of socioeconomic backgrounds, our study population reasonably reflects the general pediatric population of northern India. The methods used in our analyses are routinely available, even at medical facilities located in resource-limited settings. Hence, our results can be easily extrapolated for any establishment providing care to sick children. 

The classical hematological derangements described in dengue infection are thrombocytopenia, leucopenia, and hemoconcentration^9^ ^,^ ^10^. The majority of our patients also depicted these findings; however, a significant proportion also exhibited leukocytosis and anemia. Our study population collectively had low MCV, low MCHC, and high RDW-CV, possibly a reflection of the high prevalence of anemia in the country^11^. Girls in our study had higher rate of complications compared with boys; however, there was no significant difference in weights for age between the two sexes. The observation remains unexplained based on nutritional status and requires further exploration. We did not notice a correlation between weight for age, marker for nutritional status of children, and any of the clinical outcomes in the study. This finding is consistent with that documented by a study group in El Salvador^12^. Additionally, we did not observe any significant relationship between weight for age or BMI and disease severity among dengue-infected patients. In our study, history of bleeding from any site and presence of jaundice or palpable liver or spleen were associated with various poor outcomes. Age did not affect the outcomes. However, a report from Brazil revealed age >5 years, abdominal pain, and painful hepatomegaly as early predictors for the development of dengue hemorrhagic fever^4^ ^,^ ^13^. While we explored the role of initial hematological parameters in predicting clinical outcomes, few research groups reported contradicting results on the subject. A study from Thailand^14^ refuted any such possibility, against an observation from the Philippines^15^ that suggested the association of thrombocytopenia and hemoconcentration with the severe forms of disease. Similarly, Tanner et al.^16^ emphasized that platelet count <50,000/mL early in the illness associated with poor prognosis during the clinical course. Though, a study from Sri Lanka confirmed the results in terms of thrombocytopenia, it proposed a drastic fall in TLC early in the illness as a predictor of severe disease^17^. This contradicts our observation of higher TLC early in the disease as a predictor of worst clinical outcomes, including mortality. The results of a study from Hounduras involving 320 dengue-infected patients, using multivariable logistic regression, showed that the presence of ascites with an odd ratio of 7.29 and a platelet count <50,000 at admission with an odd ratio of 3.02 significantly associated with plasma leakage. The presence of petechiae and headache associated with better prognosis^18^. Rai et al., however, observed a poor relationship between thrombocytopenia and bleeding manifestations until platelet counts dropped below 20,000/mL^19^, in stark contrast to our results. Interestingly, in our study, the minimum platelet count recorded during the course of illness did not show any relation with the mortality.. A plausible explanation for this could be that in most children, there was an initial gradual fall in the platelet, reaching the minimum count later in the course of illness and followed by a gradual rise and clinical recovery. However, a low count early in the course of illness indicated unfavorable course during the subsequent days. A study from Thailand^20^ indicated that few abnormal laboratory findings during the febrile stage of dengue might predict for the risk of shock. Among these abnormalities were a 25% rise in Hct and platelet count <40,000/mL that predicted the possibility of shock, with relative risk ranging 4.8-10.9, similar to our findings of an odd ratio of 5.53 (95% CI: 1.90-16.09) for initial platelet count ≤20,000/mL for outcome of mortality. While we observed some debatable correlation of MPV with mortality, there is scant literature on its value as a predictor of clinical outcomes in children with dengue. A recent Indian study^21^ explored some characteristics of platelets-MPV, platelet distribution width, and platelet-crit-among patients with dengue and found no significant abnormalities compared to controls. Another Indian study^22^ examined the prognostic role of MPV in 200 patients with dengue infection and reported no significant utility. A large retrospective cohort study, based on secondary data from the epidemiological surveillance of dengue in Brazil, reported gastrointestinal bleeding, hematuria, and thrombocytopenia among the major predictors of mortality in dengue-infected patients^23^. We have proposed a cut-off of initial TLC as >7,500/mL for predicting mortality among children with dengue infection with 90% sensitivity and 70% specificity, based on the results of ROC curve analysis. Leucopenia seems to be the usual derangement in dengue infection, but a high TLC at initial evaluation may herald an exaggerated inflammatory response, characteristic of severe clinical course^6^. 

Our study fairly evaluates the proposed hypothesis and provides some simple markers for projecting clinical outcomes for children admitted with dengue infection. Although the retrospective nature of data collection is a limitation, our results provide information that may help clinicians, especially in resource-poor settings, in their decision-making process and attract researchers for further exploration of this issue.

## CONCLUSIONS

Hematological parameters observed early in the course of dengue infection may predict its clinical outcomes in infected children. Initial high TLC, low initial platelet count, and possibly low MPV are potential predictors of shock and fatal outcome in the course of disease.
